# Visual Search for Human Gaze Direction by a Chimpanzee (*Pan troglodytes*)

**DOI:** 10.1371/journal.pone.0009131

**Published:** 2010-02-09

**Authors:** Masaki Tomonaga, Tomoko Imura

**Affiliations:** Primate Research Institute, Kyoto University, Inuyama, Aichi, Japan; Queen Mary University of London, United Kingdom

## Abstract

**Background:**

Humans detect faces with direct gazes among those with averted gazes more efficiently than they detect faces with averted gazes among those with direct gazes. We examined whether this “stare-in-the-crowd” effect occurs in chimpanzees (*Pan troglodytes*), whose eye morphology differs from that of humans (i.e., low-contrast eyes, dark sclera).

**Methodology/Principal Findings:**

An adult female chimpanzee was trained to search for an odd-item target (front view of a human face) among distractors that differed from the target only with respect to the direction of the eye gaze. During visual-search testing, she performed more efficiently when the target was a direct-gaze face than when it was an averted-gaze face. This direct-gaze superiority was maintained when the faces were inverted and when parts of the face were scrambled. Subsequent tests revealed that gaze perception in the chimpanzee was controlled by the contrast between iris and sclera, as in humans, but that the chimpanzee attended only to the position of the iris in the eye, irrespective of head direction.

**Conclusion/Significance:**

These results suggest that the chimpanzee can discriminate among human gaze directions and are more sensitive to direct gazes. However, limitations in the perception of human gaze by the chimpanzee are suggested by her inability to completely transfer her performance to faces showing a three-quarter view.

## Introduction

Gaze perception is one of the most critical social–cognitive abilities possessed by primates, including humans. In humans, mutual gaze or eye contact plays a critical role in regulating social interactions by providing information and expressing intimacy (for a review, see [1]). Like humans, great apes such as gorillas (*Gorilla* spp.), orangutans (*Pongo* spp.), bonobos (*Pan paniscus*), and chimpanzees (*Pan troglodytes*) frequently exhibit social staring behavior, defined as prolonged gazing by one individual at another when both are in close proximity to each other, in various social contexts [2], [3], [4], [5]. Furthermore, studies of mother–infant pairs of chimpanzees have found that the frequency of mutual gaze increased when the infant reached about 2 months of age [6]. In contrast, many species of simian primates exhibit “gaze aversion” because mutual gaze or eye contact frequently triggers antagonistic interactions between those involved in the gazing behaviors [7], [8], [9], [10]. Although the sensitivity of apes and monkeys to direct gaze varies, these findings demonstrate that nonhuman primates can and do discriminate gaze direction. However, the cues used by these species for making judgments about being watched by others remain unclear. One reason for this lingering ambiguity is the difficulty experienced by observers in precisely identifying the target of these animals' gazes in the context of natural or semi-natural habitats (e.g., [11]).

Few empirical studies of gaze perception in nonhuman primates have been conducted in the laboratory, even though many researchers recognize the importance of gaze perception in relation to social cognition and the theory of mind [12]. Chimpanzees can follow the direction of human and conspecifics' gazes and use these gaze cues for object discrimination based on cues provided by either the eyes alone or the eyes in combination with the head orientation [11], [13], [14], [15], [16], [17], [18], [19], [20]. In addition, infant chimpanzees look at human faces with direct gazes for a longer period of time than they look at those with averted gazes when they reach the age at which they exhibit mutual gaze with their mothers [21]. A gibbon (*Hylobates agilis*) infant has also been reported to exhibit a direct-gaze preference in response to schematic faces used as stimuli [22]. Similar studies of macaques and other simian primates have also been performed. Rhesus macaques (*Macaca mulatta*) can discriminate among the gaze directions of human faces [23], [24], [25]. Like great apes, various species of monkeys also follow the directions of others' gazes and use these cues for object discrimination [14], [26], [27], [28]. In response to facial stimuli, macaques scan eye regions more frequently than they scan other regions [29], [30], [31]. However, their sensitivity to direct gaze and the effects of gaze direction on their visual behavior are rather inconsistent, although most studies agree that macaques can discriminate among the directions of the gazes of others. One study reported that infant macaques looked *less* at the direct-gaze faces of monkeys than at their averted-gaze faces, showing clear evidence of gaze aversion [9], whereas another study reported the opposite results using a human face [31]. Furthermore, both humans and rhesus macaques attended to eye regions of conspecific faces but not to the eye regions of faces of other species [32]. More recently, eye-tracking studies have found that chimpanzees also attended to eye regions, as do humans, but that their degree of fixation on the eyes was less than that exhibited by humans [33], [34].

Since the 1960s, many studies have examined how humans distinguish between direct and averted gazes [35], [36], [37]. Humans can distinguish an averted gaze when it is at least 1.4° from a direct gaze [36]. Furthermore, humans are more sensitive to a direct gaze than to an averted gaze [38], [39]. During a visual-search task, human observers detected the direct-gaze target among averted-gaze distractors more quickly than they detected the averted-gaze target among direct-gaze distractors. Such search asymmetry [40] with respect to gaze direction is known as the “stare-in-the-crowd” effect. This effect may be closely related to the direct-gaze preference in apes [21], [22] because the direct gaze captures the observer's attention in both phenomena [cf. 41].

It is well known that the morphology of the eyes of chimpanzees (and of other nonhuman primates) is quite different from that of humans [42]. This fact raises questions about what cues chimpanzees actually use during gaze discrimination. The color of the exposed area of sclera in the chimpanzee is much darker than that in humans. Humans have a dark iris and white sclera, but the sclera is darker than the iris in most chimpanzees. Such low-contrast eyes may not be suitable for the medium of visual communication frequently observed in humans, which is also consistent with the results of observational studies indicating that the great apes exhibited staring behavior when the distance separating two individuals was minimal, about 30 cm [3], [5]. Previous studies have reported that nonhuman primates can discriminate gaze directions in laboratory experimental contexts, but most of these studies have used human faces or schematic faces with high-contrast eyes [23], [24], [25], [31]. Thus, it is plausible that the primate subjects in these studies used cues different from those used in the context of their everyday lives, even though high-contrast eyes are actually processed in the same areas of their brain (i.e., the superior temporal sulcus and lateral intraparietal area), as is the case in humans [23], [24], [25], [43].

In the present study, we trained one adult chimpanzee to perform a visual-search task involving human gaze direction. This study was designed to meet two goals. The first goal was to establish whether the chimpanzee exhibited the stare-in-the-crowd effect; that is, whether she would demonstrate more efficient search for direct-gaze than for averted-gaze human faces, which are not conspecific but have high-contrast eyes. The chimpanzee participating in the present study had been raised by human caregivers from infancy but spent most of her time with other chimpanzees as well as with human experimenters and caretakers. During her extensive time with humans, this chimpanzee might have learned to discriminate among human eye gazes and their meanings. We thus hypothesized that the chimpanzee would exhibit an efficient search for a human direct-gaze face, but would also differ substantially from humans with respect to gaze processing. This was examined in Experiment 1.

Our second aim was to identify those characteristics of human eyes that were critical to the chimpanzee's ability to discriminate among gazes. This was examined in Experiments 2–4. It is clear that the position of the dark iris in the eye region serves as the discriminative cue for humans with respect to the gaze of conspecifics. Interestingly, in addition to contrast *per se*, contrast polarity (i.e., whether the iris or sclera is darker) is also crucial in judgments about the gaze direction of humans by humans [44]. Furthermore, when the brightness of the left and right sides of the sclera of a direct-gaze eye differ, the perceived gaze direction shifts to the darker side of the sclera, which is referred to as the “bloodshot” illusion [45]. If the chimpanzee identified the gaze direction on the basis of the high contrast of human eyes, as do humans, the same effect would be observed in the chimpanzee. This hypothesis was examined in Experiment 2.

We used front-view faces in the first visual search experiment. Direct gaze can be unambiguously defined for these stimuli because the iris is located at the center of the eye. However, when the head is rotated, the direct gaze should be calculated on the basis of the relationship between the position of the iris and the degree of head rotation [25]. For example, if we see the eye region of the direct-gaze three-quarter-view face separated from the facial context, we will judge that these eyes did not make eye contact with us. The so-called “Wollaston illusion” represents one of the best examples of the impact of the relationship between eye regions and face contours on the discrimination of gaze direction [46], [47]. Wollaston [47] found that a change in the orientation of the face (i.e., mirror reversal) resulted in a shift in the perceived gaze direction even though the eyes themselves remained unchanged. If the chimpanzee discriminated between the gaze directed at her (the directed-to gaze) and the gaze directed away from her, she would be demonstrating an ability to distinguish the direction of a gaze even when the stimulus head was rotated. However, if the chimpanzee simply attended to the eye region alone, her ability to discriminate would deteriorate when the faces were shifted from the direct frontal view. This was examined in Experiment 3 by manipulating the relationship between eye regions and face contours.

## Methods

### Chimpanzee Participant

Chloe, an adult female chimpanzee who was 20 years of age when the experiments began, participated in the experiments. Chloe was born in a zoo and raised by human caregivers. She was moved to the Primate Research Institute, Kyoto University (KUPRI), Japan, when she was 4 years of age. She has engaged in various types of computer-controlled perceptual–cognitive tasks, including visual-search tasks [48], [49], [50], [51]. She has also engaged in face recognition tasks using a matching paradigm [52] as well as in visual-search tasks involving the orientation of faces [53]. Before this study, she participated in an orienting task using human gazes as cues [54]. Chloe lives in a social group of 14 individuals in an environmentally enriched outdoor compound (770 m^2^) connected to the experimental room by a tunnel [55]. She was not deprived of food or water during the study, and no invasive treatments or special restraints were used in the present study. The care and use of the chimpanzee adhered to the 2002 version of the Guide for the Care and Use of Laboratory Primates by the KUPRI, which is compatible with the guidelines issued by the National Institutes of Health in the United States of America. The research design was approved by the Animal Welfare and Animal Care Committee of the KUPRI and the Animal Research Committee of Kyoto University. All procedures adhered to the Japanese “Act on Welfare and Management of Animals.”

### Apparatus

Experimental sessions were conducted inside an experimental booth designed for chimpanzees (1.8×2.15×1.75 m). A 21-inch color CRT monitor (NEC PC-KH2021) with a capacitive touchscreen device (Microtouch SM-T2) was installed 15 cm from the floor on one side of the booth. Touching the monitor surface with a finger was defined as a response. The screen was protected from deterioration by a transparent Plexiglas panel fitted with an armhole (10×47 cm) that allowed hand contact with the CRT. The resolution of the monitor was 640×400 pixels. One hundred pixels corresponded to 55 mm. Chloe sat approximately 40 cm from the monitor surface; thus, 100 pixels corresponded to 8° visual angle, and 1° corresponded to approximately 7 mm (13 pixels). A food tray was installed below the CRT monitor. A universal feeder (Biomedica BUF-310) delivered pieces of food (apples or raisins) to this tray. All equipment was connected to a personal computer (NEC PC-9821 Xn) that controlled the stimulus display, detected touches to the CRT, delivered rewards, and collected data.

### Experiment 1: Testing the “Stare-in-the-Crowd” Effect in the Chimpanzee Using the Visual-Search Task

#### Stimuli

**Figure 1:**
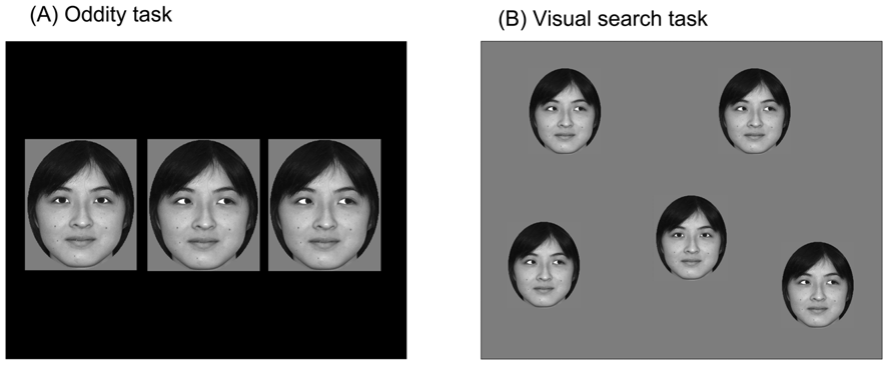
Schematic examples of the stimulus display. (A) oddity discrimination for Experiment 1 (preliminary training and transfer tests) and Experiments 2–5, and (B) visual search task for Experiment 1.

Gray-scale photographs of the front-view faces of 18 Asian women were prepared (Fig. 1). Chloe was unfamiliar with all individuals depicted in the photographs, which were retouched with Paintshop Pro® 3.0 and Photoshop® CS2 to 130×160 and 95×117 pixels in size and trimmed into elliptical shapes. Larger stimuli were used in pretraining under each condition, and smaller stimuli were used in the visual-search testing because the size of the monitor did not allow us to use the larger stimuli in the visual-search setting. The diameter of the iris was approximately nine pixels in the larger stimuli and seven pixels in the smaller stimuli, and the distance between the left and right irises was 40 pixels in the larger and 30 pixels in the smaller stimuli. Using direct-gaze stimuli as the baseline images, we prepared averted-gaze faces by shifting the iris to the right. This shifted distance was six pixels in the larger and four pixels in the smaller stimuli. These stimuli were presented on a black or gray background (Fig. 1). Note that all the individuals appearing in the photographs presented in this article provided written informed consent for their publication.

**Figure 2:**
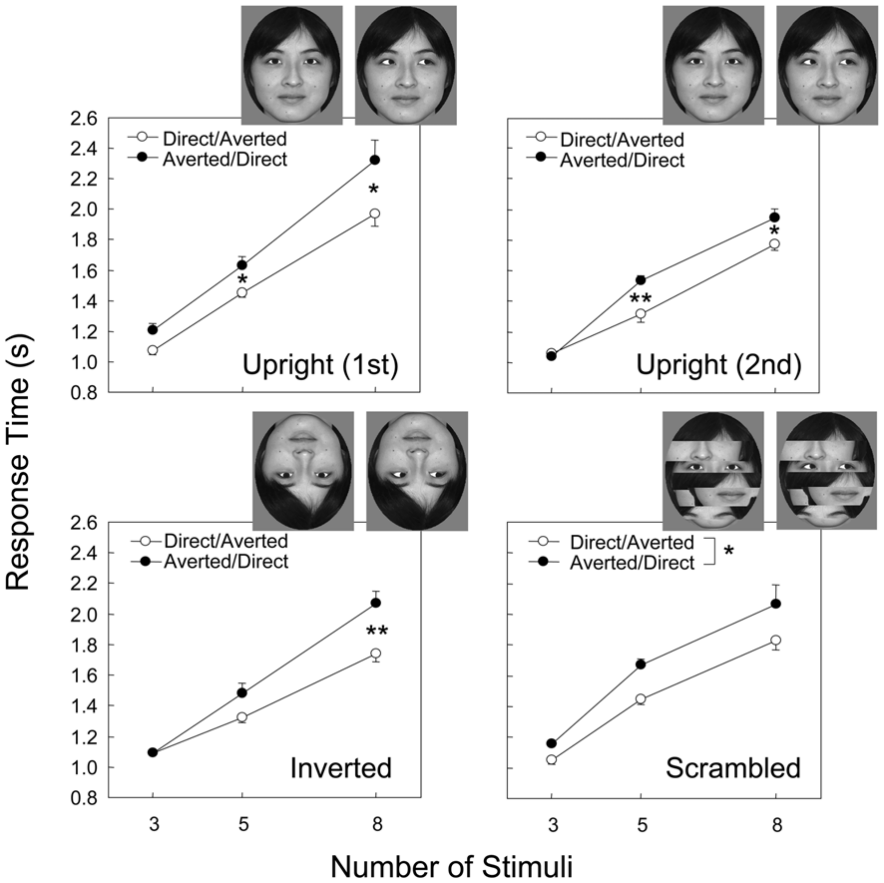
Mean response times in correct trials for each condition as a function of the number of stimulus items in the visual-search testing in Experiment 1. Error bars indicate standard errors of means across sessions. *p<0.05; **p<0.01. Chloe showed faster response times when the direct-gaze face than when the averted-gaze face was the target irrespective of the facial configurations.

We also prepared three types of faces by manipulating facial configurations. The first configuration involved an upright face, and the second involved an inverted face. The third configuration involved a scrambled face, in which the eyebrows, nose, and mouth of an upright face were randomly rearranged, while the eye positions remained intact (Fig. 2). Six conditions were prepared according to whether the direct or averted gaze served as the target and on the basis of the type of face presented.

#### Pretraining

Prior to testing with the visual-search task, Chloe participated in a 3-item oddity discrimination task as pretraining under each condition (Fig. 1A). In this pretraining, a single face was randomly selected from the 18 faces. Each trial typically began with the presentation of a 0.5-s beep sound and a blue circle (40 pixels in diameter) at the bottom center of the CRT display as a start key. When the chimpanzee touched this circle, three photographs (130×160 pixels in size) were presented horizontally. The distance between the photographs was 200 pixels from center to center. One stimulus (target) differed from the other two stimuli (distractors) in terms of the direction of the gaze depicted. If the chimpanzee touched the target, all stimuli disappeared, a 1-s chime was presented, and a food reward was delivered. If the chimpanzee touched a distractor, all stimuli disappeared, a 0.5-s buzzer was sounded, and the same trial was presented again until the correct choice was provided (correction trials). The intertrial interval was 3 s. Each session consisted of 144 trials, with the target position randomly distributed among them.

When accuracy did not improve during the course of training, we introduced two types of stimulus-fading training: an “iris position-shift training” for the direct-gaze target trials and an “iris brightness-fading training” for the averted-gaze target trials. The intact target was initially paired with a face without irises (i.e., the iris area was the same color as the sclera) in both types of training. When the rate of correct responses exceeded 80%, the iris was gradually shifted from the rightmost position to the normal averted-gaze position under the iris position-shift condition, and the darkness of the iris gradually faded into the normal direct-gaze brightness level under the iris brightness-fading condition. The pretraining session continued until the rate of correct responses exceeded 80% for two consecutive sessions under the normal condition.

#### Transfer tests

After Chloe's performance reached the criterion level, she engaged in two series of tests measuring her ability to transfer gaze discrimination to stimuli that differed from those used in the pretraining. Each transfer test consisted of four 216-trial sessions in which pretraining stimuli appeared in 144 trials (baseline trials) and new stimuli appeared in 72 trials (test trials). Six new stimuli were randomly selected from the stimulus pool. Each test stimulus appeared 12 times in each test session. The responses in each test session (the first and second series) of each trial were differentially reinforced. After the first four test sessions, Chloe was trained in gaze discrimination using the new stimuli (144–150 trials per session) until her rate of correct responses exceeded 80%; this was followed by the second test series, in which six other new stimuli were presented. The second test also consisted of four sessions.

We then selected the four stimuli associated with Chloe's best performances for use in the visual-search testing. Using the smaller-sized stimulus sets (95×117 pixels), we conducted 3-item oddity training. Chloe participated in three to six sessions of this training until her rate of correct responses exceeded 80% for three consecutive sessions under each condition.

#### Visual-search test

After the completion of the three-item oddity training using the smaller-sized stimuli, Chloe immediately began the visual-search testing. Each trial began with the presentation of the start key (blue circle, 40 pixels in diameter) at the bottom center of the CRT screen and an accompanying 0.5-s beep sound. When she responded to the start key, it disappeared, and the search display appeared on the screen. The search display consisted of a 4×2 predefined stimulus presentation area containing one target stimulus and several uniform distractors that differed from the target in gaze direction (95×117 pixels in size; Fig. 1B). The number of search items varied among three, five, and eight, and the configuration of the search display changed randomly from trial to trial. The target position was counterbalanced. The chimpanzee's task was to detect the target and touch it on the screen. Feedback accompanying the responses matched that used in the pretraining. When Chloe responded incorrectly (i.e., touched one of the distractors), the same search display reappeared. If she made a second error, only the target stimulus appeared on the screen during a second correction trial. This correction procedure was introduced to prevent Chloe from stopping her participation when the rate of non-reinforced trials was too high [48], [53].

#### Experimental design

The testing conditions proceeded in the order described in Table 1. No visual-search testing was conducted under the first condition (upright D/A  =  direct-gaze target/averted-gaze distractor with upright face), which was regarded as preliminary training. Upright D/A and A/D conditions were tested twice to verify the effects of prolonged training, but transfer tests were not conducted in the second cycle. Chloe participated in nine to 12 sessions under each condition, and data obtained in the last six sessions were used for analyses. Using SPSS 14.0J, analyses of variances (ANOVAs) were conducted on the error score and response-time data under each condition using sessions as repeated measures.

**Table 1:** Summary of the results of Experiment 1.

	Pretraining				Transfer tests (% Correct)				Small-size training		Visual search		
			Criterional Sessions^4)^		First Test		Second Test						
Condition^1)^	# of Sessions		% Correct	Response Time (s)	Baseline	Test	Baseline	Test	# of sessions	% Correct	# of sessions	% Correct^6)^	Response Time (s)^6)^
Up-D/A (pre)	18^2)^	(position [10])^3)^	89.9	0.873	92.7	50.0	87.5	75.7	-----^5)^	-----	-----^5)^	-----	-----
Up-A/D(1)	38	(dark [33])	84.4	1.206	86.8	41.3	78.4	64.2	3	87.5	9	84.7	1.736
Inv-A/D	13		87.2	1.166	79.9	75.7	85.4	83.0	3	89.6	9	82.1	1.584
Scr-D/A	36	(position [25])	86.1	1.273	89.9	75.0	81.1	71.9	5	83.2	9	85.4	1.525
Up-D/A(1)	4		92.7	0.974	93.1	81.3	91.0	81.6	4	87.2	9	92.2	1.515
Scr-A/D	26	(dark [18])	86.1	1.313	78.8	47.3	93.1	70.3	4	86.1	12	86.5	1.680
Up-A/D(2)	2		96.2	0.956	-----^5)^	-----	-----	-----	6	80.2	9	96.0	1.546
Inv-D/A	14	(position [11])	94.4	1.178	96.2	41.3	90.8	79.2	3	91.4	12	95.7	1.445
Up-D/A(2)	2		95.5	0.869	-----	-----	-----	-----	6	92.8	12	94.4	1.432

1) Up: upright face, Inv: inverted face, Scr: scrambled face, D: direct gaze, A: averted gaze. The letter before the slash designates the target and the letter after the slash designates the distractor.

2) Special fading training sessions were included in the number of sessions.

3) Type of fading training: position, shift in the iris position; dark, iris darkness fading. The numbers in the brackets show the number of fading sessions.

4) Data from the last two sessions for each condition.

5) Not conducted.

6) Data from the last six sessions.

### Experiment 2: Effects of Brightness Contrast on Gaze Perception

Experiments 2–5 used three-item oddity tasks instead of visual-search tasks. In these experiments, we examined the generalization or transfer of the discrimination performance to the sets of new stimuli instead of investigating visual-search asymmetries. Thus, we focused primarily on accuracy data in Experiments 2 and 3.

**Figure 3:**
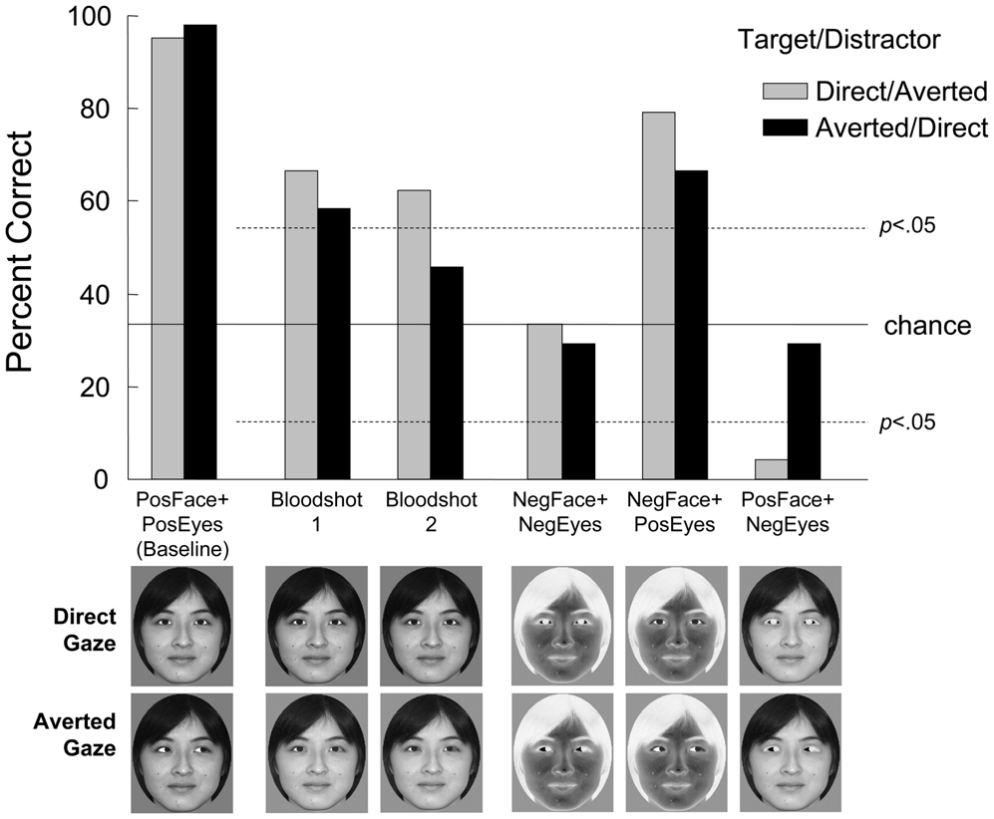
Mean accuracy in each of the baseline and test conditions in Experiment 2. Examples of the stimuli are shown below the graph. Pos: positive polarity, Neg: negative polarity. Broken lines: 5% significance levels of binomial tests for each test condition. Chloe exhibited the so-called “bloodshot illusion.” Furthermore, her behavior was controlled by the contrast polarity.

We used a new stimulus set in Experiment 2 (Fig. 3). One of the 18 stimuli used in Experiment 1 was selected and manipulated to produce the following six types of stimuli. (1) *Positive face with positive eyes*: Both the eyes and the other regions were of normal contrast polarity. The grayscale value of the iris was set to 0 (minimum value, darkest) and that of the sclera to was set to 255 (maximum value, brightest). Both direct- and averted-gaze faces were prepared and used for baseline trials. (2) *Bloodshot 1*: The direct-gaze face was identical to that of Stimulus (1), but the right part of the sclera in both eyes of the direct-gaze face was darkened to a grayscale value of 138 in the averted-gaze face because previous literature on humans as subjects showed that perceived gaze direction shifted to the side in which the sclera was darkened [45]. (3) *Bloodshot 2*: The right part of the sclera of both eyes was darkened to a greater extent than in stimulus (2), to a grayscale value of 102. (4) *Negative face with negative eyes*: The contrast polarity of both the eyes and the other regions was reversed. (5) *Negative face with positive eyes*: The contrast polarity of the eyes remained normal, but that of the other regions was reversed. (6) *Positive face with negative eyes*: The contrast polarity of the eyes was reversed, but that of the other regions was normal. These manipulations were implemented using Paintshop Pro® 3.0 and Photoshop® CS 2. The size of these stimuli was 130×160 pixels.

Test sessions of Experiment 2 were inserted between the conditions of Experiment 1. Chloe participated in two series of test sessions in Experiment 2. The first series, conducted immediately after the first upright D/A condition of Experiment 1, involved two test sessions in which the direct-gaze face was the target. The second series consisted of two test sessions in which the averted-gaze target appeared immediately after the second upright A/D condition of Experiment 1. The sequence of events in each trial was identical to that in the pretraining oddity task of Experiment 1. Each session consisted of 72 baseline trials in which a positive face with positive eyes appeared and 60 test trials in which the other five types of stimuli appeared equally often. Correct positions were counterbalanced. If the chimpanzee made an error, only the positive face with positive eyes appeared as a target in the next correction trial, irrespective of whether it was a baseline or test trial. In total, Chloe participated in 144 baseline trials and 24 trials for each type of test stimulus under both the direct-gaze and averted-gaze conditions.

Binomial tests were used to determine the statistical significance of differences between the actual accuracy rates for each type of test and those expected on the basis of chance (i.e., 33.3%).

### Experiment 3A: Tests on the Transfer to New Front-View and Three-Quarter-View Faces

**Figure 4:**
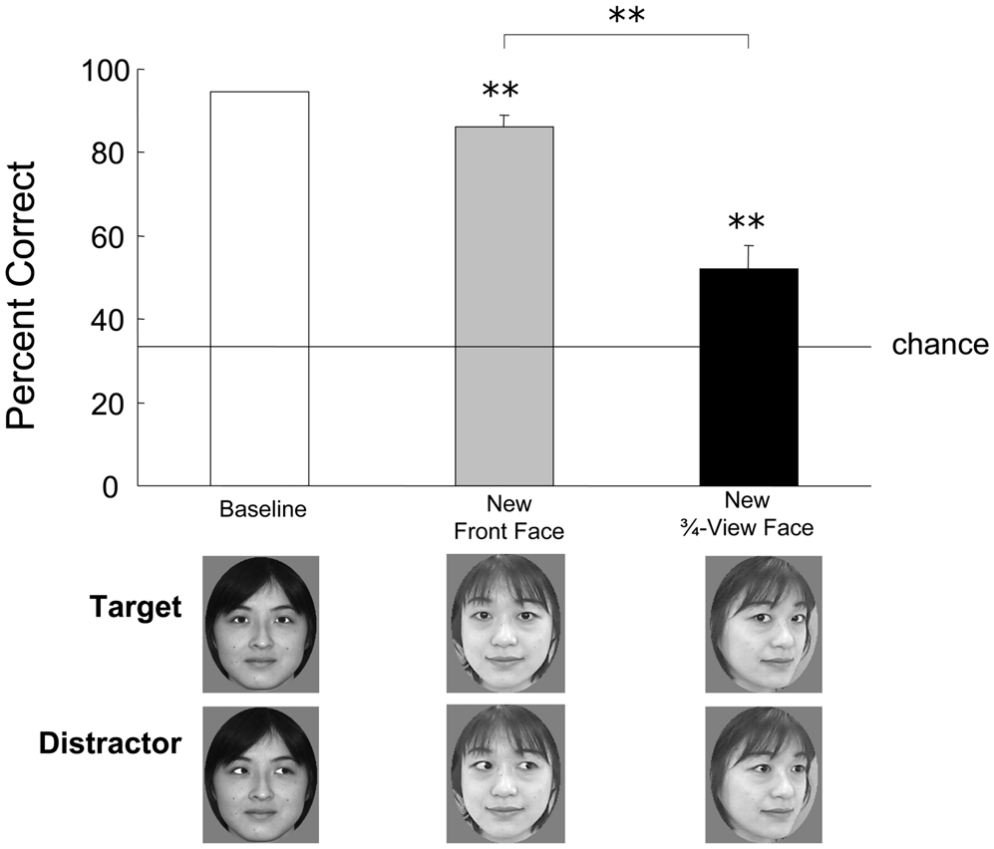
Mean accuracy in transfer tests using novel front-view and three-quarter-view faces in Experiment 3A. Examples of the stimuli are shown below the graph. Error bars indicate the standard errors of means across test stimuli. **p<0.01. The chimpanzee showed significant transfer of eye-gaze discrimination from front-view to three-quarter-view faces, but the accuracy was significantly lower than that for the new front-view faces.

Three-quarter-view faces of 10 new women were prepared for Experiments 3A–D. Three new front-view faces were also prepared. These photographs were the same size as those used in Experiment 2. These newly introduced stimuli were manipulated according to the purpose of each test series (Fig. 4–6).

**Figure 5 pone-0009131-g005:**
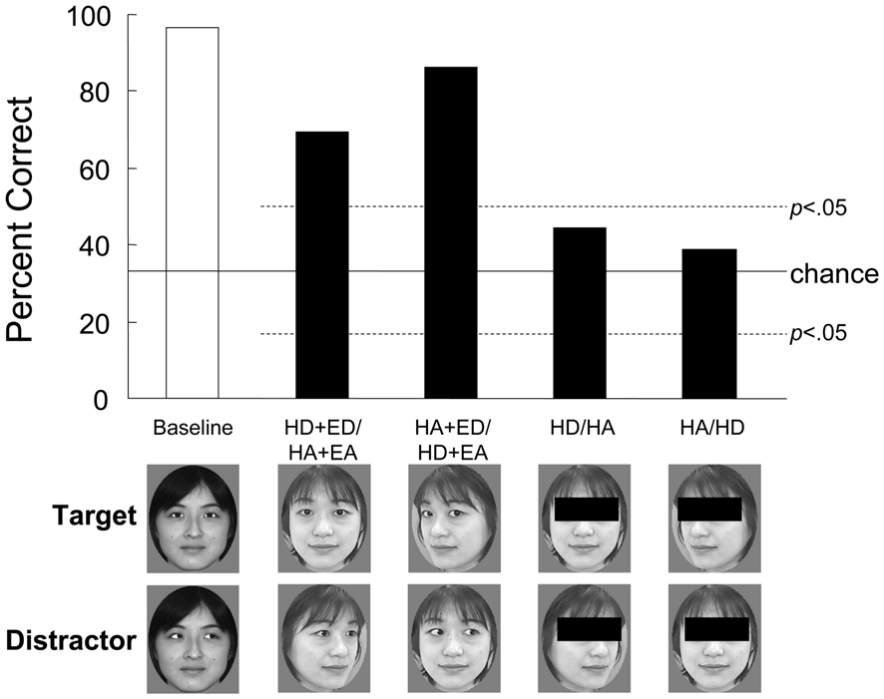
Mean accuracy in tests for the transfer to head orientation in Experiment 3B. Examples of the stimuli are shown below the graph. HD, head direct; HA, head averted; ED, eyes direct; EA, eyes averted; broken lines, 5% significance levels of binomial tests for each test condition. Chloe showed significantly above-chance performance for head-orientation discrimination, but showed chance-level performance when the eye regions were masked.

**Figure 6:**
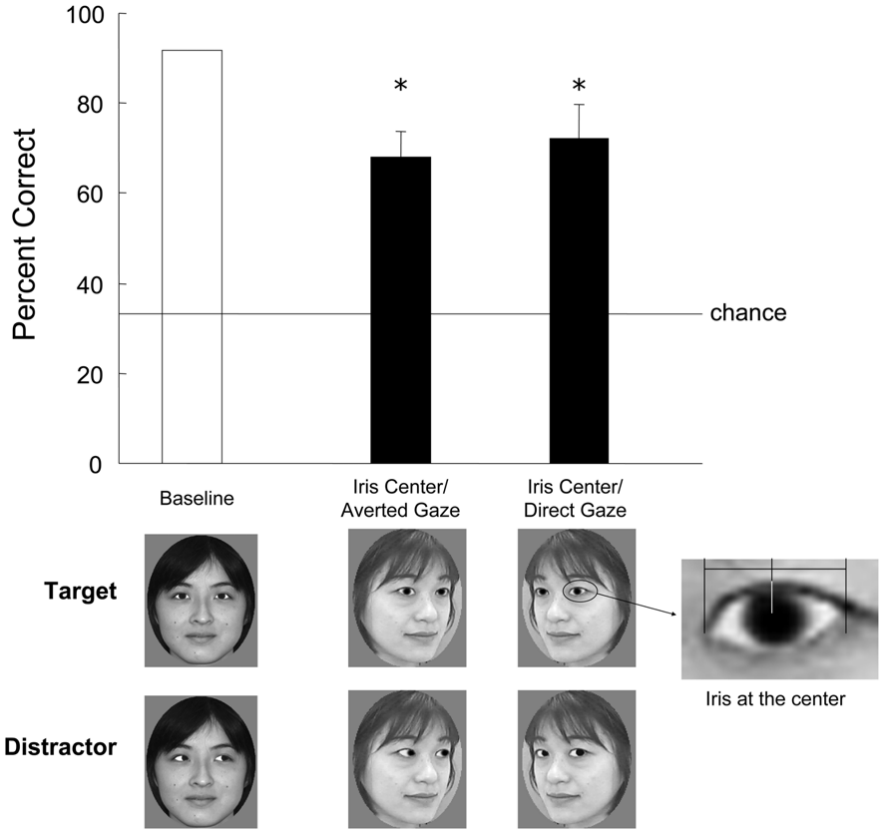
Mean accuracy for each condition in Experiment 3C. Examples of the stimuli are shown below the graph. The target stimuli contained eyes with the iris located at the center. White bar indicates the results for baseline trials, and black bars show those for test trials. Error bars indicate the standard errors of means across test stimuli. *p<0.05. The chimpanzee significantly chose the face with iris at the center of eye region more than the other types of faces irrespective of “eye contact”.

Chloe was shifted to Experiment 3 immediately following the last condition of Experiment 1 (the second test of upright D/A). The direct gaze was always the target, and Chloe participated in four successive test series. Each session consisted of 108–144 trials, depending on the type of test. The number of sessions for each test series also varied between one and 11. We used the same three-item oddity task and procedure as used in Experiment 2. Baseline and test trials were presented alternately. Baseline stimuli were the same as those used in Experiment 2. If the participant made an error, only the target appeared in the subsequent correction trials.

In Experiment 3A, Chloe participated in the transfer tests using novel front-view and three-quarter-view faces. In this test, she was tested for the transfer of discrimination ability to the three new front-view faces and the 10 new three-quarter-view faces (Fig. 4). Chloe engaged in a total of 11 sessions, with 603 baseline trials, 36 test trials with front-view faces (12 trials for each stimulus), and 270 test trials with three-quarter-view faces (12–36 trials for each stimulus). Two-tailed *t-*tests were used to determine the statistical significance of differences between the actual accuracy rates for new faces and those expected on the basis of chance as well as the statistical significance of differences between the accuracy of responses for front- and three-quarter-view faces.

### Experiment 3B: Tests for the Transfer from Eye Direction to Head Orientation

In this experiment, Chloe participated in four types of test trials using three sets of faces to test the transfer of gaze discrimination defined by eyes to that defined by head orientation (Fig. 5). (1) HD + ED/HA + EA (head direct + eyes direct/head averted + eyes averted): The target was the front-view face with direct gaze, and the distractors were the three-quarter-view faces with averted gaze. (2) HA + ED/HD + EA (head averted + eyes direct/head direct + eyes averted): The target was the three-quarter-view face with direct eye gaze, and the distractors were the front-view faces with direct eye gaze. (3) HD/HA with mask: The eye regions of stimuli used in (1) were masked with a black rectangle. (4) HA/HD with mask: The eye regions of s used in (2) were masked with a black rectangle. These two conditions were the control conditions for (1) and (2) to test the role of eye regions. These four types of test stimuli appeared equally often but randomly within a session. Chloe engaged in 216 baseline trials and 36 trials under each test condition during the four test sessions. Unlike Experiment 3A, Experiment 3B used the face of only a single individual. Thus, the results of the test trials were analyzed using binomial tests.

### Experiment 3C: Is an Iris Centered in the Sclera a Critical Cue for Gaze Discrimination in the Chimpanzee

We prepared new three-quarter-view faces based on photographs of the three people used in Experiments 3A and B. In addition to direct- and averted-gaze three-quarter-view faces, we prepared a third type of face in which each iris was located at the center of the eye (Fig. 6). We combined these three types of stimuli to produce two test conditions: *iris center/averted gaze* and *iris center/direct gaze*. Under both conditions, the target was the face with the iris in the center of the sclera. The procedure was identical to that used in Experiment 3A. Chloe received two 144-trial sessions in which the 72 baseline (target was the direct-gaze front-view face) and 72 test trials appeared in random order. If Chloe had evaluated the direct gaze on the basis of the position of the iris alone rather than on the basis of the relationship between the position of the iris and the orientation of the head, she would have chosen the three-quarter-view face with the iris at the center of the eye. The rates of correct responses under each condition (each contained three faces) were compared with those that would be obtained on the basis of chance using two-tailed *t*-tests.

## Results

### Experiment 1

#### Training and transfer tests

Eighteen sessions, including 10 special fading training sessions, were required to reach the criterion for the first pair (upright D/A) presented as preliminary training (Table 1). Overall, the number of sessions required for pretraining seemed to decrease across conditions during the course of the experiment, but this trend was not statistically significant (Spearman's test, *r*_s_ = −0.60, *N* = 9, *p* = 0.086). Not surprisingly, Chloe needed more sessions when the target–distractor mapping was reversed across conditions (28.5 sessions on average) than when the mapping was maintained (5.3 sessions on average). The mean percentage correct in the last two sessions averaged across conditions was 90.3% (standard error [*SE*] = 1.4) and the mean response time was 1.09 s (*SE* = 0.054).

During the first transfer test series, Chloe was able to achieve a 58.8% correct response rate (*SE* = 6.2) in the test trials and a 75.1% correct response rate (*SE* = 2.4) in the second test series (Table 1). Her accuracy in the test trials of both series was significantly better than would be expected on the basis of chance (i.e., 33.3%; first series: *t*(6) = 3.82, *p* = 0.004; second series: *t*(6) = 16.37, *p*<0.001, two-tailed). Furthermore, her accuracy in the second series was significantly higher than that in the first series (two-tailed paired *t*-test, *t*(6) = 2.87, *p* = 0.014), but her performance did not improve or deteriorate across conditions in the first (*r*_s_ = −0.13, N = 7, *p* = 0.788) or second (*r*_s_ = 0.14, N = 7, *p* = 0.760) series.

#### Visual-search testing

Chloe participated in nine visual-search testing sessions under five of the eight conditions, and 12 sessions under the remaining conditions (Table 1). These differences were due to the instability in the accuracy rates and response times during the earlier phase of testing under each condition. Table 2 shows the mean percentages of errors under each condition during the data-collection sessions. Overall, Chloe performed better when the target exhibited direct than averted gaze. These error data were analyzed separately for facial configurations using two-way ANOVAs (2 targets×3 stimuli). In the first set of upright faces, the main effects of target, *F*(1, 10) = 30.00, *p*<0.001, *η* ^2^ = 0.750, and number of stimuli, *F*(2, 20) = 12.62, *p*<0.001, *η* ^2^ = 0.558, were both significant. The two-way interaction was not significant, *F*(2, 20) = 0.79, *p* = 0.468, *η* ^2^ = 0.073. In the second set of upright faces, the main effect of target was not significant, *F*(1, 10) = 1.53, *p* = 0.245, *η* ^2^ = 0.133, but effect of number was significant, *F*(2, 20) = 3.88, *p* = 0.038, *η* ^2^ = 0.280. The two-way interaction was not significant, *F*(2, 20) = 0.48, *p* = 0.626, *η* ^2^ = 0.046. When the faces were presented with an inverted orientation, the main effects of target, *F*(1, 10) = 24.65, *p* = 0.001, *η* ^2^ = 0.711, and number, *F*(2, 20) = 4.83, *p* = 0.019, *η* ^2^ = 0.326, were significant, but the interaction was not significant, *F*(2, 20) = 1.03, *p* = 0.377, *η* ^2^ = 0.093. For the scrambled faces, the main effect of number was significant, *F*(2, 20) = 31.38, *p*<0.001, *η* ^2^ = 0.758, but the effect of target, *F*(1, 10) = 0.24, *p* = 0.633, *η* ^2^ = 0.024, and the interaction, *F*(2, 20) = 0.59, *p* = 0.563, *η* ^2^ = 0.056, were not significant.

**Table 2:** Mean percentages of errors for each condition during the visual search testing in Experiment1.

	Direct/Averted						Averted/Direct					
	Number of Stimuli											
Condition	3		5		8		3		5		8	
Up(1)	3.6	(0.5)	6.8	(2.0)	13.0	(2.8)	8.3	(3.1)	13.5	(2.2)	24.0	(2.8)
Up(2)	1.6	(0.7)	7.3	(2.8)	7.8	(1.9)	2.1	(0.7)	4.7	(1.8)	5.2	(1.7)
Inv	2.1	(0.7)	4.7	(1.8)	6.3	(2.6)	14.1	(1.8)	16.1	(3.2)	23.4	(4.3)
Scr	4.7	(1.8)	13.5	(2.2)	22.4	(3.2)	9.4	(2.0)	12.5	(4.0)	21.9	(3.1)

Numbers in parentheses show the standard errors of mean.

Figure 2 shows the mean response times under each condition as a function of the number of stimuli. Chloe responded faster to the direct-gaze than to the averted-gaze target, irrespective of facial configuration. The response-time data for facial configuration were also analyzed separately using a two-way ANOVA (2 targets×3 stimuli). The main effects of target *F*(1, 10) = 7.15, *p* = 0.023, *η* ^2^ = 0.417, and number of stimuli, *F*(2, 20) = 274.75, *p*<0.001, *η* ^2^ = 0.964, were significant for the first set of upright faces. The two-way interaction was also significant, *F*(2, 20) = 3.44, *p* = 0.052, *η* ^2^ = 0.256. Further tests of the simple main effects [56] revealed that the effect of the target was significant when five, *F*(1, 10) = 9.62, *p* = 0.011, *η* ^2^ = 0.490, and eight, *F*(1, 10) = 6.36, *p* = 0.030, *η* ^2^ = 0.389, stimuli were used. The main effects of target, *F*(1, 10) = 7.75, *p* = 0.019, *η* ^2^ = 0.437, and number, *F*(2, 20) = 267.22, *p*<0.001, *η* ^2^ = 0.964, were significant for the second set of upright faces. The two-way interaction was also significant, *F*(2, 20) = 3.92, *p* = 0.037, *η* ^2^ = 0.282. The effect of the target was significant when five, *F*(1, 10) = 12.91, *p* = 0.005, *η* ^2^ = 0.563, and eight, *F*(1, 10) = 4.79, *p* = 0.053, *η* ^2^ = 0.323, stimuli were used.

When the faces were presented with an inverted orientation, the main effects of target, *F*(1, 10) = 10.78, *p* = 0.008, *η* ^2^ = 0.518, and number, *F*(2, 20) = 173.66, *p*<0.001, *η* ^2^ = 0.946, as well as the interaction effect, *F*(2, 20) = 4.62, *p* = 0.022, *η* ^2^ = 0.316, were significant. *Post-hoc* tests of simple main effects showed that the effect of target was significant when eight stimuli were presented, *F*(1, 10) = 10.45, *p* = 0.009, *η* ^2^ = 0.511. The main effects of target, *F*(1,10) = 7.57, *p* = 0.020, *η* ^2^ = 0.431, and number, *F*(2, 20) = 120.56, *p*<0.001, *η* ^2^ = 0.923, were significant, but the interaction was not, *F*(2, 20) = 0.15, *p* = 0.861, *η* ^2^ = 0.015, for the scrambled faces.

Chloe was presented with successive stimulus conditions in a blocked-sessions manner. Thus, we cannot exclude the possibility that learning or training effects might have biased the results. To examine the training effect, we presented the upright-face conditions twice. Chloe maintained her efficient search for direct gaze during the second test series with upright faces (Fig. 2). We combined the first and second tests with the upright faces and conducted a 3-way ANOVA (2 repetitions×3 targets×3 stimuli) for the response-time data. The main effects for the number of stimuli, *F*(2, 40) = 539.04, *p*<0.001, *η* ^2^ = 0.964, the target, *F*(1, 20) = 13.30, *p* = 0.002, *η* ^2^ = 0.399, and the interaction between the target and the number of stimuli, *F*(2, 40) = 6.81, *p* = 0.003, *η* ^2^ = 0.254, were significant. Response times during the second test series were significantly faster than those during the first test series, *F*(1, 20) = 8.45, *p* = 0.009, *η* ^2^ = 0.297, and the interaction between number of stimuli and repetition was also significant, *F*(2, 40) = 4.01, *p* = 0.026, *η* ^2^ = 0.167. However, the interaction between repetition and target, *F*(1, 20) = 1.25, *p* = 0.276, *η* ^2^ = 0.059, and the 3-way interaction, *F*(2, 40) = 0.48, *p* = 0.620, *η* ^2^ = 0.024, were not significant. Thus, prolonged training did not seriously affect the results.

### Experiment 2

Chloe achieved a 95.1% correct response rate for the direct-gaze baseline and a 97.9% correct response rate for the averted-gaze baseline trials during the test sessions of Experiment 2 (Fig. 3). Under the bloodshot conditions, she performed significantly better than would be expected on the basis of chance under three of the four test conditions (58.3%–66.7% correct responses, *p*s<0.05, binomial tests). She performed better when the target exhibited a direct gaze than when it exhibited an averted gaze, especially during the averted-gaze trials under the second bloodshot condition (45.8%). This might be attributable to the manipulation of the averted- but not of the direct-gaze target. On average, 58.3% of Chloe's responses were correct under the four bloodshot conditions, *t*(3) = 5.55, *p* = 0.012. These results may imply that the so-called bloodshot illusion is also found in the chimpanzee. When the contrast polarity was fully reversed (negative face with negative eyes) and when only the polarity of the eye region was reversed (positive face with negative eyes), the rate of accurate responses decreased to the level of chance (24.0% on average, *t*(3) = 1.41, *p* = 0.255), and Chloe's accuracy deteriorated to significantly below the level of chance on one of the four test series (the direct-gaze target with the positive face and negative eyes, 4.2% correct, *p*<0.001, binomial test). However, when the contrast polarity of the eye region remained intact (negative face and positive eyes), her performance was significantly better than would be expected on the basis of chance, 72.9% on average.

### Experiment 3A

Chloe demonstrated significantly greater accuracy than would be expected on the basis of chance in response to the three new front-view faces, *t*(2) = 19.0, *p* = 0.003 (Fig. 4). Her performance in the test trials, in which the 10 three-quarter-view faces were presented, was also better than chance, *t*(9) = 3.40, *p* = 0.008, but was worse than that in the front-view test trials, *t*(11) = 3.24, *p* = 0.008.

### Experiment 3B

The test examining Chloe's ability to transfer what she had learned to stimuli using head orientation showed that she was able to transfer eye-gaze discrimination for the front-view faces to head-direction discrimination (Fig. 5). In contrast, when the eye region was masked, her performance dropped to the level of chance, suggesting the strong role of the eyes in gaze discrimination; that is, facial contours were not sufficient for judging the direction of the other's gaze. However, familiar stimuli (front-view faces) were paired with unfamiliar stimuli (three-quarter-view faces) in the unmasked test series. In this experimental context, the participant primarily selected the familiar target (under the HD + ED/HA + EA condition) or avoided the familiar distractor (under the HA + ED/HD + EA condition). Thus, these results may be interpreted in terms of Chloe's history of participation in experiments rather than in terms of the transfer of gaze discrimination from eyes to head.

### Experiment 3C

Under both test conditions (*iris center/averted gaze* and *iris center/direct gaze*), Chloe chose the face with the iris at the center of the sclera more frequently than she chose the direct or averted gaze (iris center/averted gaze: *t*(2) = 5.00, *p* = 0.038; iris center/direct gaze: *t*(2) = 4.27, *p* = 0.051; Fig. 6).

## Discussion

### Stare-in-the-Crowd Effect

In this study, we investigated how an adult chimpanzee, Chloe, perceived human gaze direction under visual-search and oddity-discrimination conditions. In Experiment 1, we examined the stare-in-the-crowd effect; that is, we explored whether Chloe would exhibit a more efficient search for a direct than for an averted gaze. Chloe was able to discriminate between the directions of human gazes in the oddity tasks, and the discrimination was successfully transferred to untrained stimuli. These results suggest that she did not use stimulus-specific features for gaze discrimination. More importantly, she exhibited faster response times when the target was a direct-gaze than when it was an averted-gaze face, and this effect was not explained by the specific order of training tests used in this experiment. Two additional observations should be noted. First, we generally observed an efficient search for direct-gaze faces, irrespective of facial configuration. These results are consistent with those reported by von Grünau and Anston [39], who originally found the stare-in-the-crowd effect among humans, using eye regions alone as stimuli, as well as in a gibbon infant [22], who looked longer at direct gazes than at averted gazes in upright, inverted, and scrambled faces. Our results also imply that face processing and gaze perception are relatively independent in the chimpanzee.

Second, the chimpanzee demonstrated a more efficient search pattern for the direct-gaze than for the averted-gaze faces, and her response times increased linearly as a function of the number of stimuli. In the visual-search experiments using much simpler stimuli, such as line orientations or line intersections, targets containing these visual “features” were more quickly detected than were targets without the features but with the distractor. Under the former condition, response times were very fast and did not increase, irrespective of the number of stimuli (parallel search, or “pop out”). Under the latter condition, however, response times increased linearly as a function of the number of stimuli (serial search) [40], [50]. These phenomena are frequently referred to in terms of search asymmetry. The present results, showing search asymmetry but not parallel versus serial search, are not consistent with those of search asymmetry experiments using simpler stimuli [40], [50], but are consistent with those of previous experiments on visual searches of gazes [38], [39], which have shown search asymmetry for gaze but not parallel search for direct gaze. It is noteworthy that efficient but not parallel searches have generally been reported in search experiments with humans and chimpanzees using faces as stimuli [53], [57], [58]. Our results suggest that the direct gaze did not “pop out” from among averted gazes for the chimpanzee as it did for humans, even though the processing of direct gazes may be more efficient than that of averted gazes in the chimpanzee.

### What Cues Did the Chimpanzee Utilize for Gaze Discrimination

Experiments 2 and 3 explored the cues used by the chimpanzee during the present experiments. In Experiment 2, we manipulated the brightness contrast and contrast polarity of the eye regions. When the sclera with asymmetrical brightness in both eyes was presented, the chimpanzee perceived these eyes as averted even though the irises were located at the center of the eyes. This effect is known as the “bloodshot” illusion. Our results showed clear evidence of this illusion, suggesting that chimpanzees and humans use similar kinds of cues to discriminate among the gazes of front-view faces. Furthermore, when the contrast polarity was reversed, the participant's performance deteriorated severely, as observed in humans. Both brightness contrast and contrast polarity are critical to discriminations made by humans and chimpanzees with respect to the direction of human gazes.

Experiment 3 examined the robustness of the results on direct gaze in the context of head rotations. If Chloe could categorically discriminate among the gaze directions of front-view faces—that is, if she could choose the face exhibiting “eye contact” under the direct-gaze target conditions and *vice versa*—she would have chosen the “eye-contact” face irrespective of its head orientation. The results of Experiment 3A, which showed that the ability to discriminate among eye gazes was significantly generalized from front- to three-quarter-view faces, supported this possibility. However, we should note that the transfer was significantly inferior for the three-quarter compared to the front-view faces. Experiment 3B also found that the ability to discriminate among eye-gaze stimuli was successfully transferred to the ability to discriminate among head-orientation stimuli. However, as noted, these results can also be explained on the basis of simple association. Furthermore, if Chloe's performance with respect to eye gaze had generalized to head orientation, she could have discriminated among the eye-mask conditions on the basis of head orientation alone. As Emery [8] noted, the gaze-perception system of humans is hierarchical, and the direction of the gaze of an individual is not determined simply by the direction of the eyes *per se*. Indeed, humans calculate the direction of the other's gaze on the basis of both the orientation of the head and the position of the irises in the eyes. The results of Experiment 3C clearly indicate that this was not the case for Chloe under the current experimental setting. The results showed that she primarily utilized information from the eye regions independently from information about head orientation to discriminate among gaze directions. Chloe just attended to the “iris located at the center of eye.” One reasons for this limited performance may relate to her long-term training history with front-view faces.

Based on our results, which indicate the relative independence of eye and head directions, on those of naturalistic observations of staring behaviors, which indicate that gaze discrimination occurs when very short distances separate individuals, and on considerations of the low-contrast eyes of chimpanzees, eye direction may not be as critical as head or body orientation for gaze discrimination by chimpanzees [59]. In particular, chimpanzees may not rely as much on eyes during social interactions with conspecifics as they do during interactions with humans. To test this possibility, additional systematic comparisons should be conducted on the ability of chimpanzees to discriminate between human and chimpanzee faces. Chimpanzees may differ with respect to their sensitivity to gaze-related information from the eyes and head even when human faces are used as stimuli. Humans show similar levels of sensitivity with respect to discriminating eye gaze and head orientation, with 1.4° for the eyes [36] and 1.9° for the head [60]. Unfortunately, no comparable psychophysical data on the gaze perception of nonhuman primates have been collected. Future investigations will provide more detailed information on the characteristics of gaze perception in nonhuman primates.

Finally, we tested only one experimentally sophisticated chimpanzee, who had been reared by humans but lived with other chimpanzees in a captive community. The generalizability of the current results to chimpanzees in general remains unclear. We hypothesize that most captive chimpanzees might implicitly learn to discriminate among human gazes on the basis of their long-term and extensive histories of social interactions with humans. Thus, our results can be extended, at least, to chimpanzees in captivity. Future studies to replicate and extend these results as well as testing with conspecific faces are required.
